# Filarial infection influences mosquito behaviour and fecundity

**DOI:** 10.1038/srep36319

**Published:** 2016-10-31

**Authors:** Katherine Gleave, Darren Cook, Mark J. Taylor, Lisa J. Reimer

**Affiliations:** 1 Department of Vector Biology, Liverpool School of Tropical Medicine, Liverpool, L3 5QA, UK; 2 Department of Parasitology, Liverpool School of Tropical Medicine, Liverpool, L3 5QA, UK

## Abstract

Understanding vector-parasite interactions is increasingly important as we move towards the endpoint goals set by the Global Programme for the Elimination of Lymphatic Filariasis (GPELF), as interaction dynamics may change with reduced transmission pressure. Elimination models used to predict programmatic endpoints include parameters for vector-specific transmission dynamics, despite the fact that our knowledge of the host-seeking behaviour of filariasis infected mosquitoes is lacking. We observed a dynamic, stage-specific and density dependent change in *Aedes aegypti* behaviour towards host cues when exposed to *Brugia malayi* filarial parasites. Infected mosquitoes exhibited reduced activation and flight towards a host during the period of larval development (L1/L2), transitioning to a 5 fold increase in activation and flight towards a host when infective stage larvae (L3) were present (p < 0.001). In uninfected control mosquitoes, we observed a reduction in convergence towards a host during the same period. Furthermore, this behaviour was density dependent with non-activated mosquitoes harbouring a greater burden of L1 and L2 larvae while activated mosquitoes harboured a greater number of L3 (p < 0.001). Reductions in fecundity were also density-dependent, and extended to mosquitoes that were exposed to microfilariae but did not support larval development.

Lymphatic filariasis (LF) is one of the neglected tropical diseases (NTD) and the second largest cause of permanent and long term disability worldwide^1^. An estimated 120 million human cases, across 55 countries, lead to a loss of 5.9 million disability-adjusted life-years (DALYs)^2^,^3^,^4^.

Three species of filarial nematodes are responsible for causing LF; *Wuchereria bancrofti, Brugia malayi*, and *Brugia timori*^4^. Transmission of filarial worms is indirect, with parasites developing within a mosquito vector before being passed to definitive vertebrate hosts. Larval development occurs within the short lived mosquito and once transmitted to a vertebrate host, parasites carry out their longer life stages and reproduction^5^. Several different mosquito species from the *Culex, Anopheles, Aedes* and *Mansonia* genera can transmit LF^4^ but susceptibility to parasite infection varies between species^6^,^7^,^8^.

An understanding of LF transmission dynamics is crucial for the implementation and monitoring of elimination programmes^9^,^10^,^11^,^12^,^13^,^14^. Mathematical models are being used to guide decision making on the best strategies to eliminate lymphatic filariasis^15^. Slight changes in vector specific parameters can alter the likelihood of elimination and the most suitable approach to reach transmission breakpoints^13^. Important parameters of the vectorial capacity equation, such as the host encounter rate, gonotrophic cycle length, host preference and vector death rate, are based on the parasite-naive vector population. However, previous research in other disease systems has shown that infection status can influence vector physiology and behaviour, and alter these important determinants of transmission.

Studies on malaria have shown that sporozoite positive mosquitoes probed more frequently and for longer periods of time^16^. These results were complemented by field studies showing that naturally infected, sporozoite-positive mosquitoes fed on a significantly greater number of hosts^17^ than uninfected mosquitoes. Anderson *et al*.^18^ further showed that host-seeking behaviours differed between mosquitoes infected with developing oocysts and the infective stage sporozoites. In this study sporozoite-infected mosquitoes were more persistent and experienced greater host contact. While it is well known that parasite infection is associated with changes in host behaviour in ways that favour onward transmission, few studies are able to definitively show that such changes are adaptive^19^. Such changes may be due to the pathological response to infection, they may be parasite-mediated or host-mediated.

Results from Cator *et al*.^20^ demonstrated that exposed mosquitoes were more responsive to host cues when the infective sporozoite stage of the parasite was present, as opposed to the developmental oocyst stage. However a similar response was observed in mosquitoes inoculated with heat-killed *Escherichia coli*, suggesting the response is a generic infection response rather than parasite mediation. Further work by this group showed that the behavioural changes were linked to changes in insulin signalling and resource-based constraints of immunity, blood feeding and reproduction. Regardless of the mechanism, reducing risky host-seeking and feeding activities during parasite developmental stages will increase the chance of mosquito survival, and hence successful parasite transmission^21^.

Further research on the behaviour and physiology of filariasis infected mosquitoes is needed to determine the capacity of vectors to sustain transmission though elimination efforts. The aim of this study was to determine how infection with *Brugia malayi* influences mosquito behaviour in the presence of host cues and the fecundity of mosquitoes. We evaluated convergence towards a human host in mosquitoes exposed to low and high densities of *B. malayi* microfilariae. We correlated the intensity of infection at the developing (L1/L2) and infective (L3) stages with the outcome in the assay and the number of mature eggs produced. We also compared the outcome between uninfected control mosquitoes and exposed mosquitoes that failed to establish an infection.

## Results

### Mean intensity and parasite yield

Mosquito infection was summarised using the mean intensity for microfilariae ingested, developing larvae (L1/L2) and infective larvae (L3) (Table 1). L1 were first detected after one day post exposure (DPE), L2 detected from five DPE and by 11 DPE 100% of all recovered filarial worms were infective L3 stage (Supplementary Fig. S1). Midgut removal confirmed that mf were escaping from the midgut and into the haemocoel within the first eight hours.

### Short range host assay

The short-range host assay measures orientation and flight towards a human volunteer. The outcome measure is the proportion of mosquitoes in the cage where host cues were present out of the total number released, here termed ‘host convergence’.

**Figure 1:**
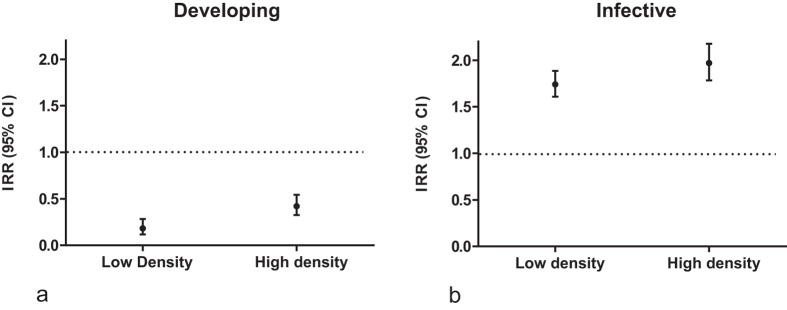
Incidence rate ratio (IRR) of mosquito convergence in the presence of host cues. (**a**) Converging mosquitoes at the developing stage (4–6 DPE) compared to the control cohort. (**b**) Converging mosquitoes at the infective stage (11–13 DPE) compared to the control cohort. All observed behaviours were significantly different than the control un-infected mosquitoes at both time points (p < 0.0001). Control (n = 790), Low Density (n = 250), High density (n = 930).

Results from the short-range host assay showed that mosquitoes exposed to *B. malayi* exhibited significantly different behaviour in the presence of a host than those fed uninfected blood (Fig. 1). During the developing time period, mosquitoes were less likely to converge on the host (low density: 14.5% [95% CI ± 5.9] converging; high density: 29.3% [95% CI ± 3.7] converging) compared to the controls (67.7% [95% CI ± 4.4]). However during the infective time period, exposed mosquitoes were more likely to converge on the host (low density: 69% [95% CI ± 9.1] converging; high density: 78% [95% CI ± 4.3] converging) than controls (43.1% [95% CI ± 5.2]) (Supplementary Fig. S2). While exposed mosquitoes exhibited significantly increased host convergence behaviour (p < 0.0001) during this period, control mosquitoes exhibited a significant decrease (p < 0.0001).

Next, we determined whether behaviours differed in mosquitoes exposed to *Brugia* but where infection failed to establish. The behaviour of mf-exposed but uninfected mosquitoes at 4–6 DPE was comparable to the control cohort with 71% converging towards host cues (n = 28) compared to 68% (n = 440) (p = 0.84). However, the behaviour of the exposed but uninfected mosquitoes at 11–13 DPE was lower with 16% converging towards host cues (n = 19) compared to 43% in the control group (n = 350) (p = 0.029).

**Figure 2:**
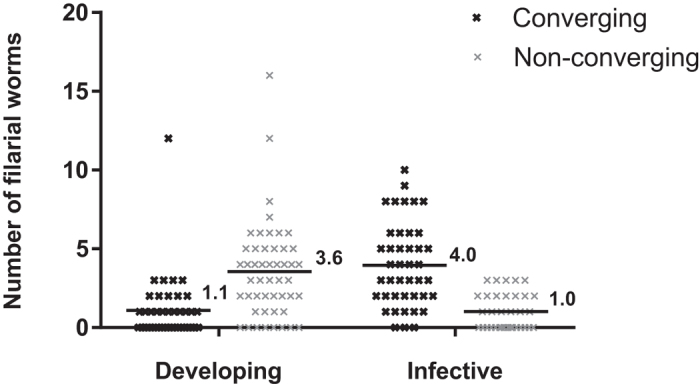
Worm burden in mosquitoes that converged on a host and those that remained in the holding cage at developing (4–6 DPE) and infective (11–13 DPE) life stages. Dark lines show mean worm burden of converging and non-converging mosquitoes.

Following the behavioural assay, 166 mosquitoes were randomly sampled (83 from the host attraction cage and 83 from the release cage across both time points) for assessment of infection prevalence and intensity. Infection prevalence was 69% during the developing stage and 74% during the infective stage, showing no significant difference. Mean worm burden was compared between converging mosquitoes from the host cage and non-converging mosquitoes from the release cage, at both the developing and infective time points (Fig. 2). During the developing time period, non-converging mosquitoes had a significantly higher worm burden than converging mosquitoes (4.0 [95% CI 3.2: 4.8] and 1.1 [95% CI 0.6: 1.9] respectively, p < 0.001). However during the infective time period, converging mosquitoes contained significantly more worms than non-converging (3.6 [95% CI 2.8: 4.5] and 1.0 [95% CI 0.7: 1.5 ] respectively, p < 0.001).

### Fecundity

**Figure 3:**
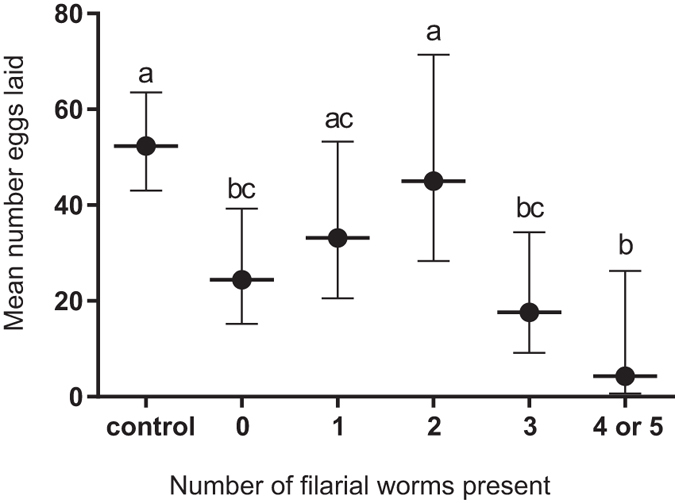
Mean number of mature eggs, including those laid or mature but free inside the abdomen, in unexposed mosquitoes (n = 20) and *B. malayi* exposed mosquitoes (n = 52) with 0–5 larvae present. Only 2 mosquitoes carried 5 larvae and neither of these had developed eggs therefore categories 4 and 5 where combined for statistical analysis. All mosquitoes had mated. Results with the same letter above the bar are not statistically significant from each other.

The mean number of eggs laid per mosquito was compared to the number of filarial larvae present in 20 control females and 52 exposed females (Fig. 3), with a significant difference overall between groups (p < 0.001). There was a significant difference between the number of eggs laid between the control mosquitoes and those that were exposed but had no worms present (mean egg number: control 52.4 [95% CI 43.1: 63.6]; exposed but no worms 24.4 [95% CI 15.2: 39.3] p < 0.05). The difference between the control cohort and mosquitoes harbouring one or two filarial worms was not statistically significant (mean egg number: one worm present 33.2 [95% CI 20.6: 53.3]; two worms present 45 [95% CI 28.4: 71.4]) There was a significant difference when three, four or five worms were present within the mosquito (mean egg number: three worms present 17.6 [95% CI 9.1: 34.3], four or five worms present 4.3 [95% CI 16.1: 32.5], p < 0.05).

### Survival

There were no significant differences in mosquito survival between the control and exposed groups. Total survival ranged from 80–93% at 16 days post-exposure, with no notable differences in worm burden between dead or moribund mosquitoes (mean worm burden = 2.9) or mosquitoes surviving through the assay (mean worm burden = 22, p = 0.4).

### Blood meal volume

There was no statistical difference in blood meal volume, measured as concentration of haemoglobin (mg/ml), between mosquitoes fed on either uninfected control blood, or those fed on blood containing microfilariae (mean haemoglobin concentration 267.6 [95% CI 237.5: 297.6]; 274.5 [95% CI 248: 301] respectively, p = 0.7015).

## Discussion

Parasite transmission is not only reliant on vector survival, but also on the ability of the vector to locate a host and feed^22^. Previous work on the effect of a *Plasmodium* infection on mosquito behaviour has shown a difference in behavioural characteristics such as host-seeking and probing^16^,^18^,^21^. These alterations in host-seeking behaviour appear to be stage-specific, with mosquitoes positive for infective sporozoites being more likely to initiate probing, probe for longer and feed to repletion. These results suggest that mosquito behaviour may be altered in order to reduce risky behaviour, such as host seeking foraging and blood feeding, when parasites are still developing, while promoting or increasing these behaviours when infective parasites are present. However subsequent work from Cator and colleagues^20^,^23^ suggested that the change in receptivity and host-seeking behaviour was a generic response to exposure, corresponding with *Plasmodium* developmental stages. Hence we investigated whether analogous behavioural change occurred in filarial-infected mosquitoes and whether this was related to the development stage of the parasite.

Our study demonstrated that female mosquitoes fed on infected blood were up to 5 times less likely to converge on a host hand when the developing stages of *B. malayi* were present. Conversely, mosquitoes harbouring infective third-stage (L3) larvae showed a significantly greater convergence in the presence of a human host compared to controls. Control mosquitoes exhibited reduced convergence between the two time points which may have been due to mosquito senescence. Previous studies have shown that senescence can influence a variety of activities such as mortality, blood feeding and flight ability, all of which affect host-seeking behaviour^24^,^25^,^26^. This suggests that the enhanced behaviour in the presence of host cues in infective mosquitoes and absence of behaviour in infected mosquitoes might be separate processes that overcome the age-related changes in behaviour. Among exposed mosquitoes we observed significant differences in the mean worm burden of converging and non-converging mosquitoes at both time points, with heavy infections associated with non-convergence during the developing period and convergence during the infective period. This suggests that both suppressive and enhanced behavioural changes are density dependent.

The mechanistic cause of the change in behaviour could be a direct parasite mediated and stage specific dependent mechanism that could drive a suppression of mosquito behaviour during the development phase of the infection to protect the parasites from the risks associated with blood feeding, but that is reversed by infective stage parasites to facilitate their transmission to the definitive host. Alternatively, it could be due to an indirect infection response (e.g. immunological or physiological), that temporally coincides with infection-related processes that vary according to the developmental stage of the parasite. Another indirect mechanism may be related to tissue damage caused by the parasite, including mf damage to the gut wall, or developing or infective larval damage to thoracic musculature or the mouthparts. Up to 30% of mf-exposed mosquitoes were negative for filarial larvae at the time of the host assay, although over 95% of mosquitoes examined one day post-exposure were found with microfilariae in the haemocoel. These mf-exposed but free from developing or infective larvae mosquitoes suffered a fitness cost with significantly fewer mature eggs, though there was no difference in host convergence behaviour compared to the uninfected cohort during the developing time period. During the infective time period, this uninfected cohort showed significantly less convergence in the presence of a host than the controls, which was the opposite pattern observed in mosquitoes with L3 present. This suggests that inoculation with microfilariae, without further viable parasite development, was not sufficient to influence this aspect of host-seeking behaviour. Cator *et al*.^23^ showed that immune challenge was sufficient to elicit the behavioural response seen in infected/infective mosquitoes, only when immediately following a blood meal. Further work is needed to confirm whether the presence of microfilariae in the haemocoel following a blood meal, rather than the presence of developing larvae and infective larvae in the thorax or other body parts, is causing the differences in convergence behaviour observed in our study.

A number of limitations exist when conducting behavioural assays in a laboratory environment with colonized mosquitoes. In this study we observed significant differences in convergence when mating and egg-laying were enabled (Supplementary Fig. S2). Previous studies on the behaviour of infected mosquitoes have not confirmed mating or enabled egg-laying, and the magnitude of differences observed in other studies may have been influenced by possible atypical physiological states of the females mosquitoes used.

We monitored mosquito survival throughout the assay in order to determine whether mosquitoes used in the L1/L2 time point may have had a higher worm burden than those used at the L3 time point due to reduced survival in heavily infected mosquitoes. In nature, filariasis vector survival is known to be affected by parasite density^8^, however the strain of *Aedes aegypti* used in our study has been selected for susceptibility to *Brugia malayi*. With no significant differences in mortality between the various cohorts, and no significant differences in mean worm burden of dead and moribund mosquitoes, we conclude that density dependent mortality had no impact on our assay results.

Fecundity experiments carried out within a laboratory setting also have limitations, as mosquito oviposition is influenced by many external and environmental factors including light levels, substrate choice and the presence of conspecific eggs or larvae^27^. For this reason we measured egg maturation, and included mature and free eggs within the abdomen as well as eggs laid in our analysis. Host fecundity reduction is a common outcome of parasite infection in insects^28^,^29^,^30^,^31^,^32^. This could be due to direct nutrient competition by developing parasites or indirect competition by draining energy reserves required for an immune response^27^. In this study the mf-exposed and infective as well as mf-exposed but without larval development cohorts both showed a decrease in the mean number of eggs produced. The reduction in fecundity was independent of variations in blood meal intake, which was similar in both groups. This suggests that exposure to infection, possibility due to immunity, may have additional fitness costs, which might influence vector physiology parameters in models.

## Conclusions

Filariasis transmission models are based on parameters that describe host-seeking behaviour, physiology and vector-parasite interactions. However, as these traits can differ between uninfected, mf-exposed, developing infections and infective populations, in a density-dependent manner, the variability in the parameter estimates could have an impact on the validity of model predictions for elimination endpoints. This study shows that stage-specific behaviour change occurs in mosquitoes infected with *B. malayi*, with increased convergence towards a host when the infective L3 stage is present and decreased convergence when the developing stage is present. Changes in fecundity among exposed and infective mosquitoes demonstrates that there are additional fitness costs related to mf-exposure as well as high density infections. While mass drug administration is reducing community-wide microfilariae prevalence and intensity, the success of the elimination programme will also depend on the ability of mosquitoes to survive and transmit filariasis under these changing conditions. Further work is needed to determine to what extent these patterns of behaviour change in host-seeking, extend to wild vectors of filariasis and how the dynamic complexity of these behaviour changes contribute to transmission dynamics as the endpoint of elimination targets are reached.

## Materials and Methods

### Mosquito rearing and maintenance

*Aedes aegypti* Liverpool strain (LVP strain) mosquitoes were reared from eggs to adults in the insectary at the Liverpool School of Tropical Medicine (LSTM) under standard conditions (27 °C and 80% relative humidity). Eggs were transferred into plastic rearing trays (23.5 × 34.5 × 7.5 cm) filled with distilled water. Second instar larvae were split between fresh trays to achieve a lower larval density, and then maintained on Chinchilla pellets. Pupae were collected, transferred into cages (28.5 × 29.5 × 28 cm) and allowed to emerge as adults. All adult males were removed one day prior to exposure, and remaining females maintained on 10% sugar solution. This rearing ensured all mosquitoes were of the same strain and age when tested.

Our preliminary studies showed that the opportunity to lay eggs significantly influenced the outcome in the short range host assay (Supplementary Fig. S3) with a fivefold increase in host convergence behaviour in females that were allowed to lay eggs. Therefore, for all of the experiments in this study we confirmed that females had mated, by examining the spermathecae of 10 females per cohort for the presence of motile sperm, and all were given the opportunity to lay an egg batch after the blood meal. A flowchart that summarises the experimental design of the below assays is included (Supplementary Fig. S4).

### Aedes aegypti exposure to Brugia malayi

Mosquitoes were split into different treatment cohorts and allowed to feed on either uninfected human blood to be used as controls or with human blood containing *B. malayi* parasites at different densities. Mf viability was confirmed through the preparation of a wet blood slide to observe motility. Microfilariae (mf) were recovered by intraperitoneal lavage from gerbils^33^. All experiments were performed in accordance with Home Office (UK) requirements. Approval was obtained for all animal experiments from the ethical committees of the University of Liverpool and LSTM. The recovered mf were diluted 1/100 in RPMI media (Sigma-Aldrich), 10 μl was added to a slide (in triplicate) and mf were counted to determine their concentration in the peritoneal lavage solution. Mf were added to human blood (obtained from the Royal Liverpool Hospital blood bank) to try and achieve the target concentrations of 7,500 mf/ml (low density) or 15,000 mf/ml (high density). Control blood had the relevant amount of RPMI media added to match that added to the infected blood when adding the mf. The actual microfilaremia in the blood samples fed to mosquitoes was calculated at the time of feeding and confirmed in triplicate. 2 drops of 2% formaldehyde solution was added to 20 μl of blood to lyse red blood cells. Using phase microscopy (x10 objective) the entire slide was scanned and all mf counted. In this way different mf densities of low (5,450–7,750 mf/ml) and high (10,550–15,400 mf/ml) ranges could be compared.

4–6 day old female mosquitoes were starved of sucrose for 18 hours prior to blood feeding with infected or non-infected blood. 3 ml of blood was offered using a Hemotek membrane feeding system. Each cohort of mosquitoes only received a single blood meal for the entirety of the study. Mosquitoes were allowed to feed for half an hour after which only fully engorged individuals were selected for all future experiments. Control mosquitoes were from the same rearing cycle and received uninfected blood to feed on at the same time as those receiving infected blood.

### Mosquito dissections to recover and observe filarial parasites

Dissections and assays were based on the average *B. malayi* development times within the mosquito (Supplementary Fig. S1). All filarial worms recovered were included in the study and recorded as mf, L1, L2 or L3 along with the body region in which they were recovered. Developing worms (L1 and L2) are present from 3 DPE, and from 11 DPE infective L3 are present.

#### Midgut dissection

To estimate the number of mf ingested at different blood microfilaremic densities, 3–5 mosquitoes were removed 4 hours after exposure and knocked down briefly on ice, to avoid damage or death to filarial worms. Intact midguts were removed and lysed in two drops of 2% formaldehyde. The number of mf present within the midgut and body was counted under x10 magnification and recorded as the number of mf ingested.

#### Abdomen and thorax dissection

Performed from <24 hours post exposure to 10 DPE. Following standard protocol, the thorax and abdomen were removed, teased apart and cover slipped to allow for the examination of mf, L1 and L2 life stages of the parasites by phase contrast microscopy.

#### Full body dissection

Full body dissections were performed 11 DPE to examine L3 stage larvae, as they were often found in the head and mouthparts of the mosquito but also observed in the haemocoel of the thorax and abdomen. Each body region was teased apart into 3–4 sections and left for 1 minute to observe motile L3 under a dissecting microscope. The mosquito was further teased apart and scanned to check for ay tissue-bound L3. Finally, the tissue was covered slipped and scanned under a phase microscope at x10 objective to look for developing larvae.

### Mosquito survival

Unexposed (n = 240 across five replicates) and exposed (n = 239 across five replicates) mosquitoes were held in separate cages and the day of death recorded. Moribund mosquitoes were removed from the cage for dissection and the day of death was classed as the subsequent day. Otherwise mosquitoes were dissected on the day they died, with dissection type according to DPE, and the number of parasites present and developmental stage noted. This experiment was concluded for each exposure at 16 DPE.

### Short range host assay

To assess the impact of infection on mosquito behaviour in the presence of host cues, a short range host assay, based on a behavioural assay used by Cator and colleagues^20^, was carried out on mosquitoes that had been exposed to *B. malayi* at 4–6 DPE (developing larvae are present within the mosquito, Supplementary Fig. S1) and 11–13 DPE (infective larvae present, Supplementary Fig. S1), followed by dissections. Control mosquitoes (n = 790), mosquitoes exposed to low density microfilariae (n = 250) and high density microfilariae (n = 930) were starved of sucrose for 18 hours prior to assay. Ten mosquitoes per replicate were released into a mesh holding cage that was 17 cm × 17 cm × 17 cm. A tube 12 cm in diameter and 48 cm in length connected this cage to another one of the same dimensions where the experimenter placed their hand 2 cm from the mesh wall (Supplementary Fig. S5). Mosquitoes were allowed to settle in the holding cage for 15 seconds. A barrier between the holding cage and the tunnel was opened for 240 seconds before closing. Mosquitoes that remained in the holding cage were labelled non-converging while mosquitoes that had landed in the second odour cage were labelled converging. There were no instances of mosquitoes returning to the holding cage after entering the odour cage, however, we did observe a few mosquitoes briefly enter the tunnel and return to the holding cage, but all mosquitoes were found in one of the 2 cages at the end of the experiment. Either five or ten batches were assayed per day at each time point, with an equal number of control and infected mosquitoes assayed. Infected and uninfected cohorts were rotated to take into consideration any changes in the time of day while testing was taking place. Mosquitoes from both the converging and non-converging group were removed for dissection as described above (n = 83 from each group) to determine whether parasites were present. A subset of mosquitoes (Control blood n = 5, Infected blood n = 5), where held individually after blood feeding to ensure that oviposition would occur before the short range host assay that started 4–6 DPE.

### Fecundity assay

Control (n = 20) and *B. malayi* exposed (n = 52) females were isolated 3 days after blood feeding for the fecundity assay. RPMI media was added to control blood in the same volume as the infected blood source, with all mosquitoes feeding at the same time. Females were placed into individual holding cups (9 cm diameter × 8 cm), covered with fine netting and provided with an oviposition cup. Mosquitoes were maintained on 10% sucrose. The cotton wool in the oviposition cups was regularly moistened with water so that it remained favourably wet for egg laying. Maintenance remained the same until death, when they were removed and the number of eggs that had been laid, if any, were counted. Ovary dissections were performed to view any eggs remaining inside the body cavity. Eggs were classified as mature if they were laid, or if they were free within the body, while immature eggs were not included in the analysis. We chose to include fully mature but retained eggs because numerous environmental and social factors may influence the deposition of eggs onto a substrate^34^.

### Blood meal volume

Blood meal volume ingested by mosquitoes fed on infected and non-infected blood (Control n = 10, Infected n = 10) was determined by quantification of total haemoglobin content in the abdomen using a colorimetric assay and Drabkin’s Reagent (Sigma-Aldrich). Blood was prepared as described above with control blood containing RPMI media and both cohorts were fed at the same time. Abdomens of these mosquitoes were immediately homogenised in 1 ml Drabkin’s reagent supplemented with Brij 23 solution (Sigma-Aldrich) and then cleared in a centrifuge for 15 minutes at 13,400 × g. Samples were loaded onto a 96 well plate and absorbance read at 540 nm using Epoch plate reader and Gen5 software. A standard curve was prepared using known concentrations of haem and haem content of experimental samples was calculated by applying the formula obtained from the standard curve.

### Statistical analysis

Statistical analysis was carried out using STATA statistical package and graphs were prepared using GraphPad Prism 6. The numbers of exposed mosquitoes that converged in the presence of host cues was compared to the control group using a Poisson regression model with robust standard errors. Adjustment for clustering between days and with total numbers of mosquitoes in each assay included as an offset variable. The robust standard error accounts for the over dispersion in this dataset. The clustered analysis by day was chosen to remove other sources of variance due to ontogeny or experimental conditions on the day the assay was conducted. The incidence rate ratio with 95% confidence intervals is presented for each time period, in relation to control mosquitoes.

The effect of worm burden on mosquito convergence towards a host, and mean number of mature eggs was analysed using Poisson regression models with robust standard errors. Blood meal volume was analysed using a paired t-test with 95% confidence intervals. Survival curves were compared using Log-rank (Mantel-Cox) comparison.

## Additional Information

**How to cite this article**: Gleave, K. *et al*. Filarial infection influences mosquito behaviour and fecundity. *Sci. Rep.* **6**, 36319; doi: 10.1038/srep36319 (2016).

**Publisher’s note:** Springer Nature remains neutral with regard to jurisdictional claims in published maps and institutional affiliations.

## Supplementary Material

Supplementary Information

## Figures and Tables

**Table 1:** Mean intensity of ingested microfilariae, developing larvae (L1/L2) and infective larvae (L3).

Mf density	Mean mf ingested (0.5 DPE)	Mean developing L1/L2 (4–6 DPE)	Mean infective L3 (11–13 DPE)
Low	14.9 ± 3.3 (n = 7)	3.0 ± 0.7 (n = 17)	1.4 ± 0.2 (n = 18)
High	23.7 ± 1.4 (n = 29)	2.6 ± 0.3 (n = 135)	3.0 ± 0.3 (n = 77)
