# Supplementary material for: Filarial infection influences mosquito behaviour and fecundity
Source: Sci Rep. 2016 Oct 31;6:36319. doi: 10.1038/srep36319 (PMC5087081; doi:10.1038/srep36319)
Supplement: Supplementary Information [file srep36319-s1.pdf]

Supplementary Information

Filarial infection influences mosquito behaviour and fecundity

Katherine Gleave[1], Darren Cook[2], Mark J Taylor[2], Lisa J Reimer*[1,2]

[1]Department of Vector Biology, Liverpool School of Tropical Medicine, Liverpool, L3 5QA, UK

[2]Department of Parasitology, Liverpool School of Tropical Medicine, Liverpool, L3 5QA, UK

*Corresponding author Lisa.Reimer@lstmed.ac.uk

S1. Filarial worm development.

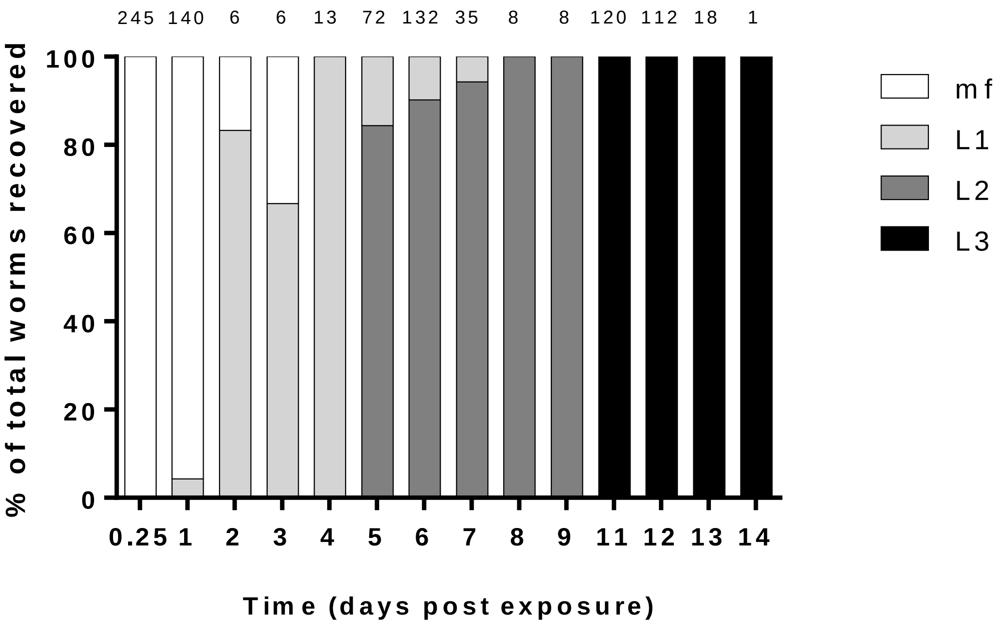

Figure S1. The development of *Brugia malayi* from microfilaria to infective stage larvae in *Aedes aegypti*. The total number of worms recovered is listed above each bar. (0.25 DPE n= 11, 1 DPE n= 12, 2 DPE n= 4, 3 DPE n= 8, 4 DPE n= 10, 5 DPE = 46, 6 DPE n= 82. 7 DPE n= 28, 8 DPE n= 3, 9 DPE n= 2, 11 DPE n= 50, 12 DPE n= 38, 13 DPE n= 4, 14 DPE n= 1).

S2. Mosquito convergence on a host when infected with developing (L1/L2) and infective (L3) stage filarial worms

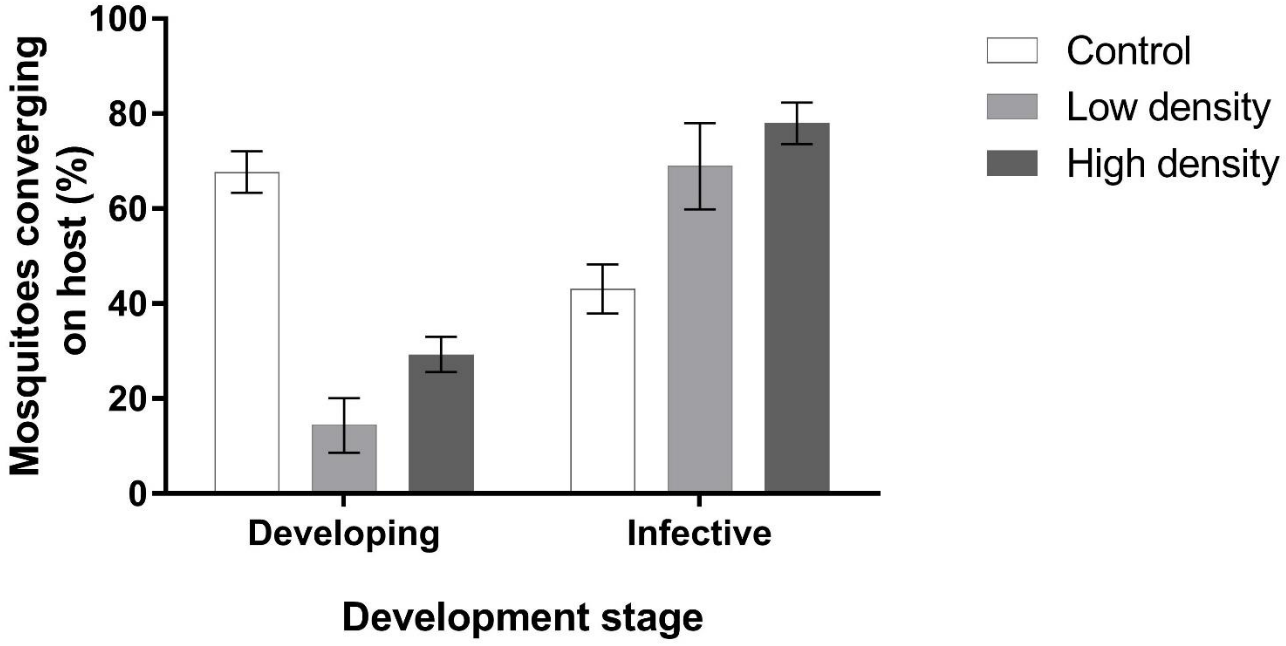

Figure S2. The percentage of mosquitoes converging on a host when exposed to uninfected and infected blood during two time points. A short range host assay was performed at developing (4-6 DPE) and infective (11-13 DPE) time points to assess the activation and orientation towards a human hand. Control (n=790), Low density (n=250), High density (n=930).

S3. Short range assay to assess the effect of oviposition.

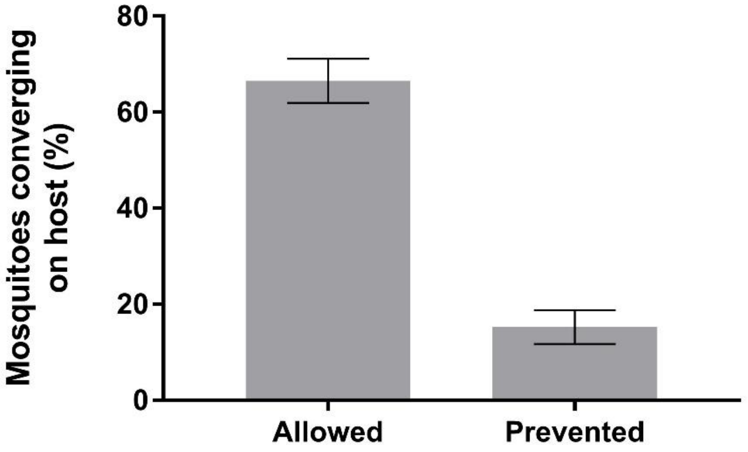

Figure S3. The percentage of mosquitoes converging on a host when oviposition was allowed or prevented. The short range assay was carried out on 2 groups of mosquitoes: one where oviposition was allowed by the addition of an oviposition substrate (n= 400), another where no substrate was provided (n= 400), to determine whether standard husbandry practices influenced activation and orientation towards a host. 6 day old females were offered a blood meal and after 3 days an oviposition cup was placed into one of the cohort cages, while the other did not receive one. Mosquitoes were sugar starved for 12 hours prior to the short range assay. This study involved 40 replicates of 10 females each per cohort. Assays were performed over 4 days, with cohorts assayed in alternate succession. We observed significantly greater activation and orientation in mosquitoes that were allowed to lay a batch of eggs after consuming their first blood meal ($P < 0.0001$ n = 400 per cohort). (Odds ratio 11.03 95% CI [7.831, 15.54]). Dissections from a subset of mosquitoes (10 per cohort) showed that 100% of females had mated.

S4. Flowchart of methodology

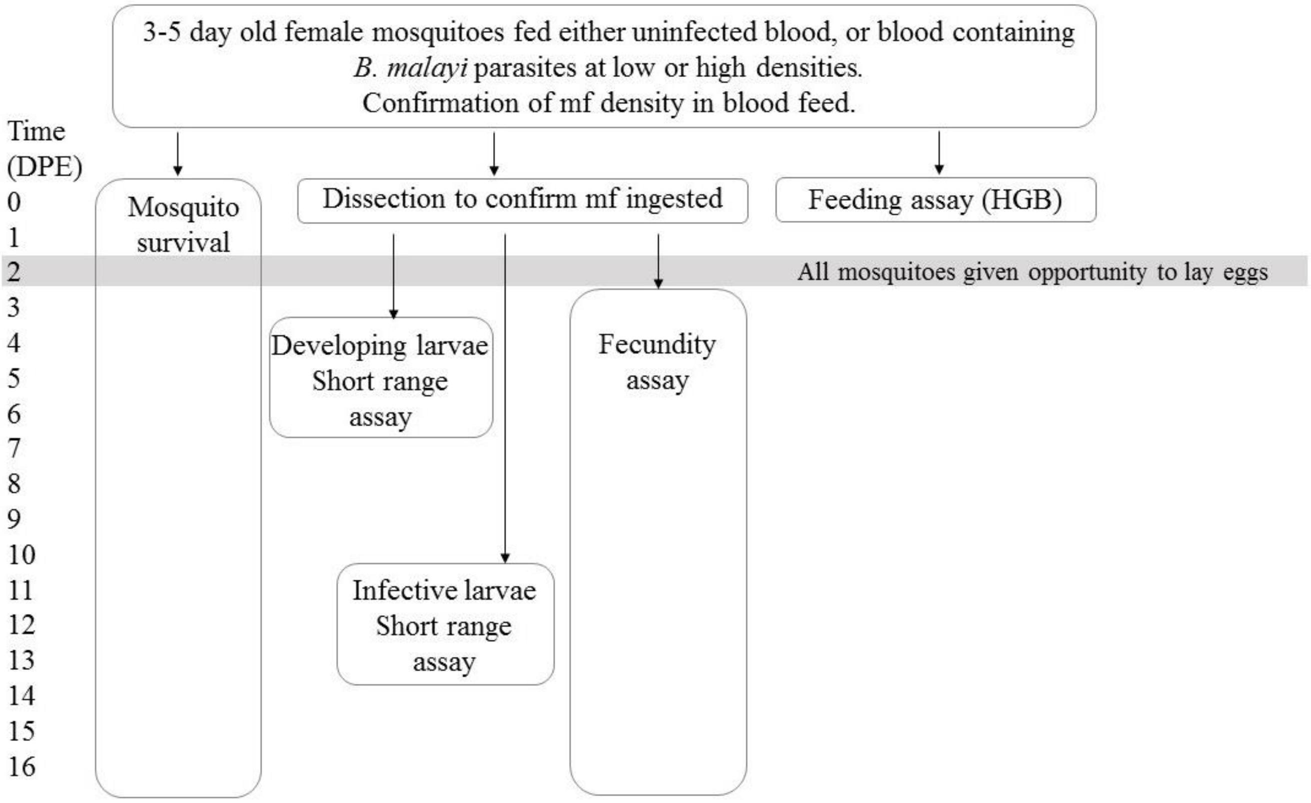

Figure S4. Flowchart of methodology. Flowchart showing structure of methodology and mosquito designation for each assay.

S5. Diagram of behavioural assay

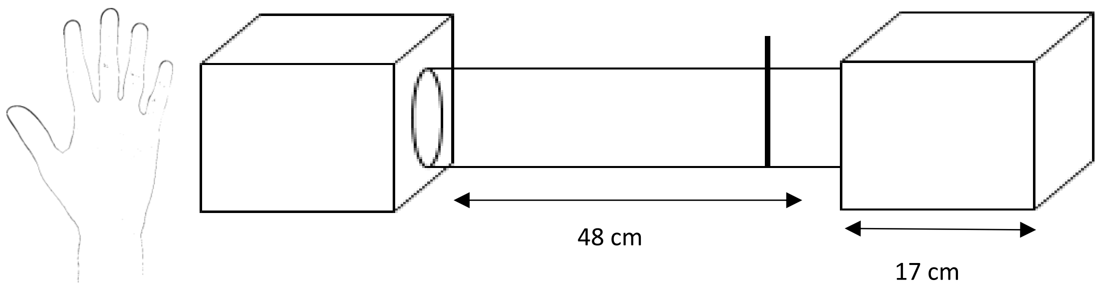

Figure S5. Experimental set-up for behavioural bioassay. A schematic of short range behavioural assay. Mosquitoes were released into a mesh holding cage that was 17cm x 17cm x 17cm. A tube 12cm in diameter and 48cm in length connected this cage to another one of the same dimensions. A gate prevented movement from one cage through to the other until the assay was ready to begin.
